# Advanced Temperature-Control Chamber for Resistance Standards

**DOI:** 10.6028/jres.125.012

**Published:** 2020-04-10

**Authors:** Shamith U. Payagala, Alireza R. Panna, Albert F. Rigosi, Dean G. Jarrett

**Affiliations:** 1 National Institute of Standards and Technology,Gaithersburg, MD 20899, USA

**Keywords:** air-flow simulations, measurement, standard resistor, temperature-control chamber

## Abstract

Calibration services for resistance metrology have continued to advance their capabilities and establish new and improved methods for maintaining standard resistors. Despite the high quality of these methods, there still exist inherent limitations to the number of simultaneous, measurable resistors and the temperature stability of their air environment. In that context, we report progress on the design, development, and initial testing of a precise temperature-control chamber for standard resistors that can provide a constant-temperature environment with a stability of ± 6 m°C. Achieving this stability involved customizing the chamber design based on air-flow simulations. Moreover, microprocessor programming allowed the air flow to be optimized within an unsealed chamber configuration to reduce chamber temperature recovery times. Further tests were conducted to improve the stability of the control system and the efficiency of the chamber.

## 1 Introduction

The “Metrology of the Ohm” project at the National Institute of Standards and Technology (NIST) is responsible for accurate calibration of resistance standards ranging from microohms (µΩ) to teraohms (TΩ). The expanded uncertainties, describing deviations within 2σ of the mean, of these measurements vary from 0.04 µΩ/Ω to 100 µΩ/Ω across more than twenty decades of resistance. Several factors influence the accuracy and the repeatability of these measurements, and among them, constant temperature control during measurement remains one of the more critical factors required to produce results with the lowest possible uncertainties [1–6].

**Fig. 1:**
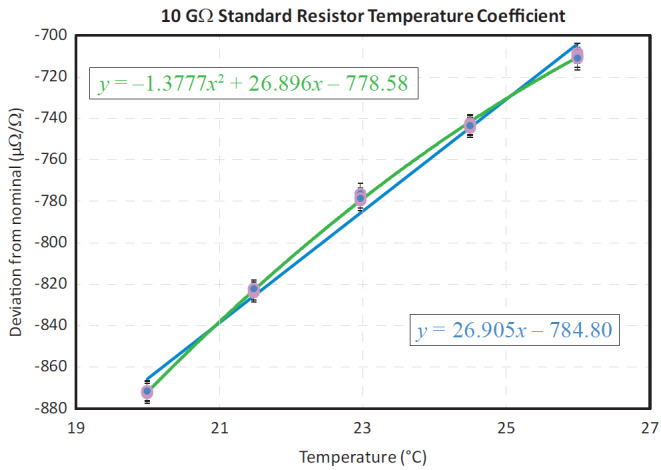
Temperature-dependent deviation for an example 10 GΩ standard resistor. The deviation from its nominal value can be characterized in two ways. In the first, and more approximate method, the trend is described linearly, with its fitted slope being equivalent to the temperature coefficient of the resistor. In the alternative approach, a more accurate quadratic fit is used to extract the alpha (linear) and beta (quadratic) temperature coefficients of the resistor.

Figure 1 illustrates the behavior of the deviation a 10 GΩ resistor from its nominal value with respect to temperature. Based on the graph, we can deduce the significant effects that environmental factors, such as temperature, can have on high-value resistors. As a result, high-value resistance standards are kept in air chambers during measurements. Air chambers are used for high-resistance measurements as opposed to oil baths because in the latter case, the resistivity of the oil allows leakage paths to ground that are significant for resistances on the order of 10 MΩ or greater. In Fig. 1, it is evident that temperature fluctuations, even those within 0.02 °C, have an effect of approximately 0.6 µΩ/Ω on the measurement result. Therefore, in order to achieve lower uncertainties, air chambers should have the ability to control their temperature to 0.01 °C or better.

There are several air chambers maintained within the “Metrology of the Ohm” project that are capable of precisely controlling temperature. However, the necessity for a new temperature-controlled chamber emerged when the size limitation of a temperature chamber used for ultrahigh-resistance measurements restricted the number of standard resistors that could be measured simultaneously.

In this work, we report progress on the design, assembly, software development, and initial testing of a precise temperature-control chamber that can provide a constant-temperature environment with a stability of ± 0.006 °C for standard resistors. Designed to fit inside a 1.5 m wide instrument rack, the chamber uses a structural frame design that includes insulated aluminum double-wall construction. Aluminum double-wall construction with 5 cm thick polyisocyanurate rigid foam insulation effectively provides thermal isolation from the laboratory environment. Cooling inside the chamber is provided by constant thermoelectric heat transfer to the outside environment. The cold air flow out of the thermoelectric device is passed through a proportionally controlled nickel-chromium heating element, and mixing occurs under a platform inside the chamber. The regulated air is then circulated in the chamber using fans mounted on space-efficient channels. For precision control, the temperature in the chamber is monitored by two thermistors with calibration interchangeability of ± 0.05 °C.

Fluid-flow simulations within the chamber were performed during the design phase to model air flow that would optimize gradient reduction. Effective placement of the input and output fans was identified as a critical factor to eliminate gradients within the chamber. Several other items such as adequate insulation, system fail-safe mechanisms, and reduction of chamber settle times were also addressed. The first item was addressed by adjusting the insulation thickness and thermal insulation material.

The second and third items are more closely related to the chamber settling time, specifically, after unsealing the chamber when switching resistors. An improved control mechanism based on a microprocessor and a graphical user interface (GUI) was implemented to attain user-friendly control and automation of the chamber. The microprocessor constantly monitors the temperature, set point, fan speed, and overall status of the temperature chamber. Furthermore, the microprocessor manages the air flow within the chamber while the door is open to lessen chamber temperature recovery times.

Special tasks such as temperature coefficient measurements for standard resistors require an advanced monitoring and control functionality that can communicate with the measurement system control software. Remotely controlling the chamber temperature allowed for the automation of temperature coefficient measurements like those shown in Fig. 1. After the chamber was assembled, additional tests were conducted to verify the stability of the control system and the efficiency of the chamber.

## 2 Air Bath Design from Numerical Simulations

### 2.1 Fluid-Flow Simulations

**Fig. 2:**
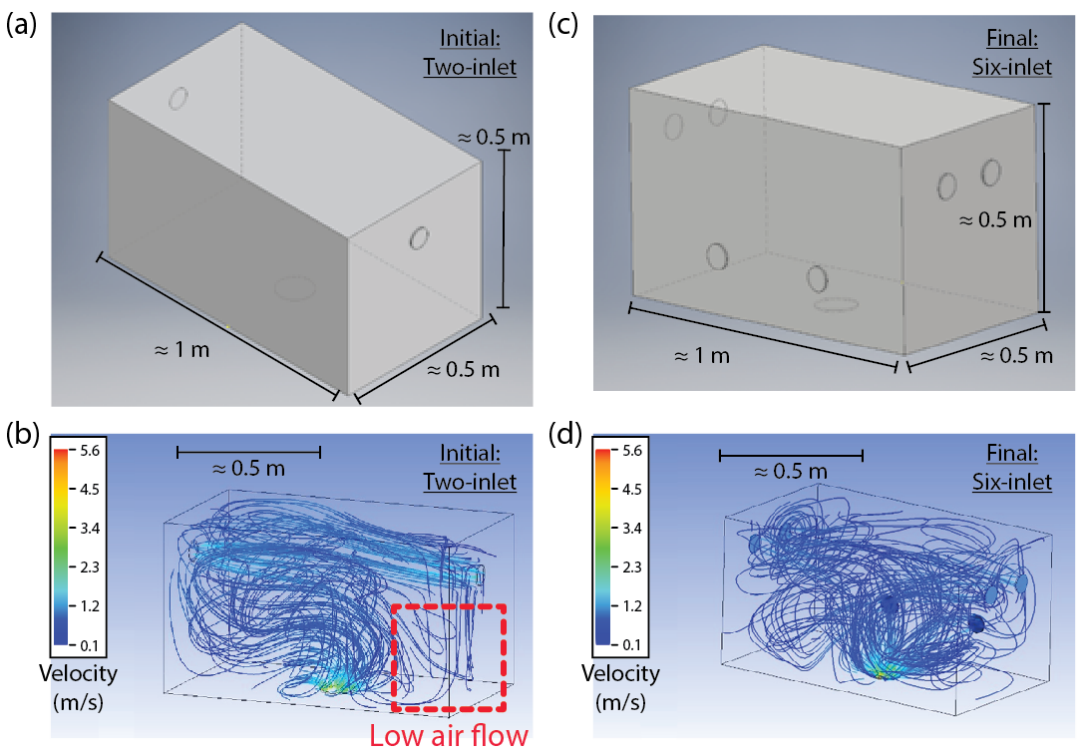
(a) Initial CAD model of the inner chamber. (b) Fluid-flow simulation with the initial CAD model. A significantly large region of lower air flow is marked by the dotted red box and contributes to potentially larger temperature gradients within the chamber. (c) Modified CAD model of the inner chamber. (d) Fluid-flow simulation with the modified CAD model, demonstrating the elimination of large regions with lower air flow. This elimination promotes a more homogeneous temperature within the chamber.

Initially, the usable space inside the chamber was modelled by computer-aided design (CAD) software. Figure 2(a) shows the initial CAD design, where the output air fan is placed on the bottom of the chamber, and the input air fans were arbitrarily placed on two parallel walls of the air chamber, both orthogonal to the bottom wall. The thermoelectric cooling device, which is discussed in a later section of this article, included a fan with volumetric flow rate of 0.035 m^3^/s. Placement of the thermoelectric device was limited to the bottom surface of the inner chamber due to the size of the unit and proximity to the mixing chamber, the function of which will be clarified in the next section. However, during initial design, it was assumed that input air fans would not have a location constraint.

To construct an effective air bath chamber that exceeds current-day limitations on size, and thus possible number of simultaneous resistor measurements, investigation of the temperature gradients within the chamber was crucial. Fluid-flow simulations were performed to obtain solutions for the technical issues encountered during this design phase, removing the need to rely on expensive and time-consuming trials. An additional advantage to performing simulations was the ability to make modifications to the design in real time [7]. The simulations were performed with Fluid Flow (CFX) from the ANSYS software package.^1^1 Certain commercial equipment, instruments, or materials are identified in this paper to foster understanding. Such identification does not imply recommendation or endorsement by the National Institute of Standards and Technology, nor does it imply that the materials or equipment identified are necessarily the best available for the purpose. The Fluid Flow software not only accommodated our numerical model for optimizing placement of fans and structural elements, but it also allowed a visual random-particle representation of the expected air flow.

After the three-dimensional (3D) model of the inner chamber was designed, ANSYS software was used to simulate the air flow of input and output fans [8–15]. Starting with the fundamental Navier–Stokes equation, physical processes like heat, momentum, and mass transfer can be characterized, with *ρ*, *v*, *P, τ,* and *g* being the air density, air velocity, air pressure, stress tensor (representing air viscosity), and Earth’s gravity, respectively:

$\frac{\partial }{\partial t}\left(\rho v\right)+v\cdot \nabla \left(\rho v\right)=-\;\nabla P\;+\;\nabla \cdot \tau +\;\rho g$. (1)

Equation 1 is generally obtained when assuming conservation of mass. CFX uses a finite-volume approach to solve partial differential equations and, for our purposes, also requires both boundary and initial conditions. Because gravity only contributes values four to five orders of magnitude smaller than those contributed by the total pressure *P*, it may be neglected for a small volume. One of the more crucial features of CFX is its ability to solve both fluid-flow and pressure parameters by coupling them as a single system, ultimately resulting in fewer iterations required to achieve solutions that converge properly. We considered the case of turbulent flow given that the relevant Reynolds number for this system is at least on the order of 10^5^, in part due to the lower air velocities and smaller physical dimensions, and this number is not far below the expected onset of turbulent flow for flat boundaries. Adjusting this estimation for flow near the fans only increased the Reynolds number, justifying our assumption of turbulent flow (and subsequent use of the standard *k*-ε model).

To accurately simulate the behavior of the temperature chamber, the simulation software required the user to predefine the inlets, outlets, and boundaries of the system. Several key characteristics such as mass flow rate based on the volumetric flow values, flow direction, and the fluid material were taken into consideration during the program setup. For the steady-state simulation, initially, the volumetric flow value of an arbitrary DC brushless fan was used for the input air fans, whereas the volumetric flow value of the output air fan on the bottom surface was left unconstrained, as warranted by the thermoelectric cooler device chosen for the system. In order to simulate the air flow of the fans, volumetric flow was converted to mass air flow based on the pressure, temperature, and relative humidity parameters of air. For the calculation’s initial parameters, standard atmospheric pressure of 101.325 kPa, room temperature of 23 °C, and relative humidity of 50% were used. By extension, it was assumed that air density remained constant. These mass flow rate values were then entered to corresponding fans within the simulation. Approximately 10^4^ nodes and 4.5t × 10^4^ elements were used. The system had a convergence criterion based on a residual root mean square less than 2 × 10^−4^.

Simulation data, as shown in Fig. 2, parts (b) and (d), were carefully analyzed to determine the ideal locations for the input fans and their volumetric flow ratings. Upon visually inspecting the first simulation in Fig. 2(b), it became evident that the flow rate at the level of the fans appeared to be much higher than that at lower heights where fans were not present. In light of this, reallocation and addition of fans were considered. Six fans, placed in the configuration shown in Fig. 2(c), were used for the subsequent simulation. Fans on the left and right were placed higher than the fans on the back surface to achieve uniform flow within the chamber. Flow simulations performed for this modified CAD model are exemplified in Fig. 2(d). The modified model, bearing similar parameters and conditions to the initial simulation, demonstrated a more uniform flow throughout the chamber. Despite modeling both cases as turbulent flow, we found that turbulence intensities were much lower than 2% for regions several centimeters away from the fans, suggesting that these regions away from any boundaries or fans could perhaps be modeled with laminar flow. Several trial and error simulations were performed using characteristics of commercially available fans to determine the ideal volumetric flow value for the fans.

**Fig. 3:**
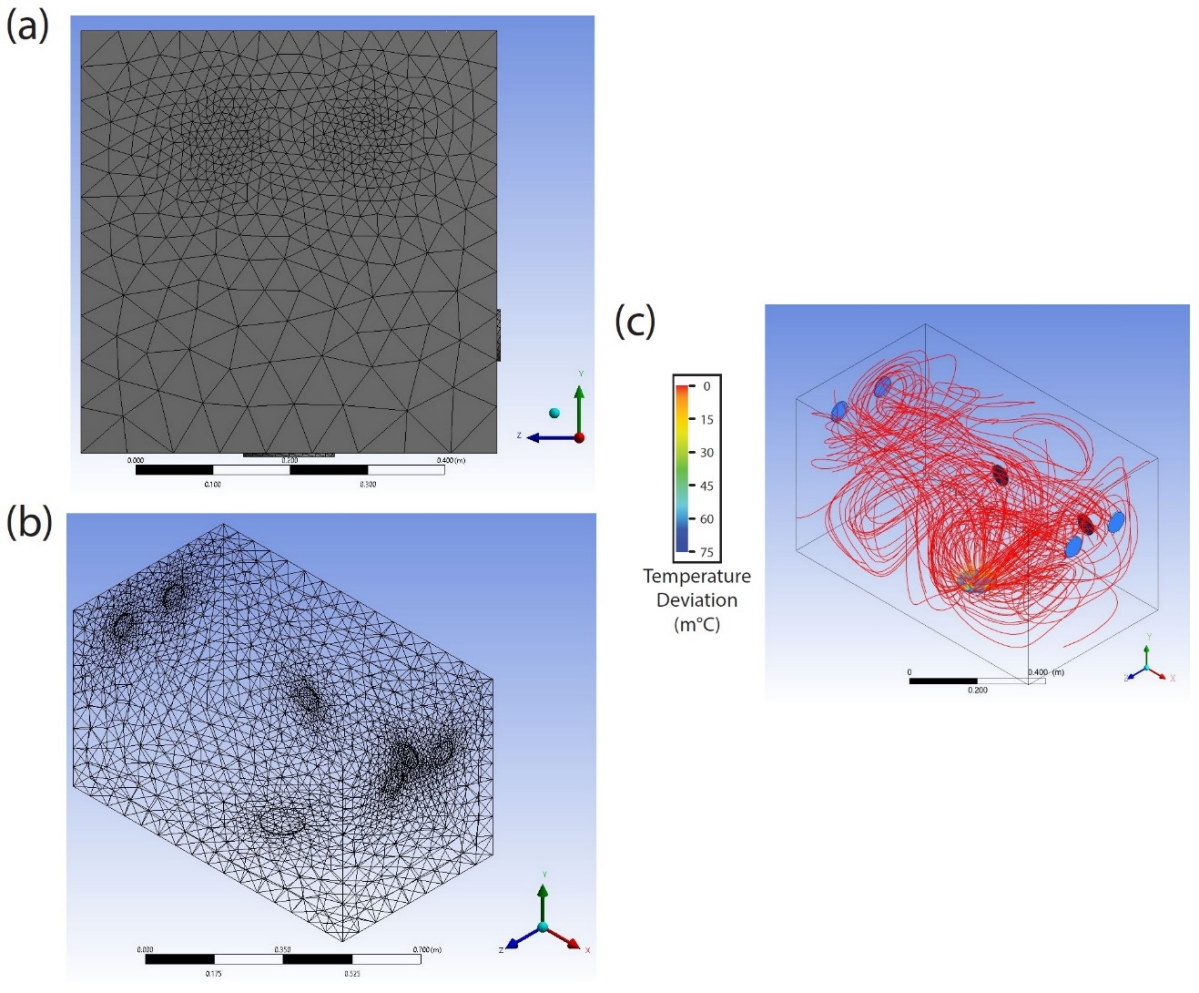
(a) Final CAD model meshing of the inner chamber, with the view on one of the sides with two inlets. (b) Full view of the final CAD model meshing of the inner chamber. (c) Results of the temperature distribution within the inner chamber. Fans are colored black and blue as a guide to the eye for back wall and side walls, respectively.

For this modified model, examples of the mesh are provided in Fig. 3, parts (a) and (b), highlighting the importance of increased mesh density by the inlets and outlet. In this same model, the resulting temperature gradient is shown in Fig. 3(c) and appears to show very little deviation from the initial condition. Even with temperatures very close to the outlet, where flow velocities reach a maximum, deviations were simulated to be less than 0.015 °C.

### 2.2 Structural Design and Assembly

The computational fluid dynamics (CFD) design verification then allowed for advancement to the structural design and assembly phase. Design parameters that needed consideration included space efficiency, weight, ease of assembly, and adequate insulation, and all implementations were done with 3D modelling software. The software also provided flexibility for design changes, allowed for easy testing of structural integrity, and calculated the usable space within the chamber.

The final design of the chamber was partially determined by constraints in its eventual location within the laboratory. Thermal isolation from the laboratory environment was achieved using an aluminum double-wall design that consisted of 5 cm thick polyisocyanurate rigid foam insulation. The thickness was selected based on past data gathered on laboratory conditions. Specifically, the figure of merit used to determine insulation was the RSI value, a measure of how well a two-dimensional (2D) barrier resists heat flow (RSI indicates the International System of Units representation of the *R-*value, a number frequently used in the building industry for thermal insulation). In our case, the final value was 2.11 m^2^·K/W.

**Fig. 4:**
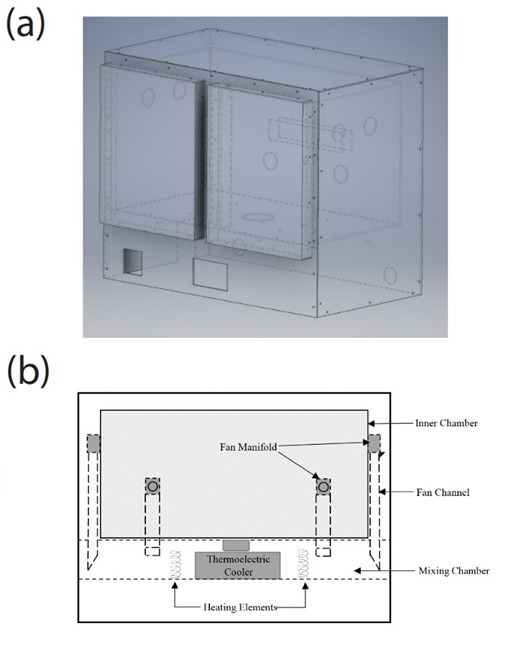
(a) 3D model of the final chamber design. A space-efficient design was adopted to eliminate excess weight while maintaining structural integrity. (b) Diagram of the inner chamber, which includes the mixing chamber and its components.

The aluminum double wall design was achieved using 4.7 mm thick plates of aluminum bolted to the structural frame. An inner chamber, with inner dimensions of 96 cm (length), 54 cm (width), and 53 cm (depth), was also fabricated using 4.7 mm thick plates of aluminum, which were mounted to the inside of the structural frame, allowing 5 cm of empty space between the outer plates and the inner box. Rigid foam insulation was placed between the outer aluminum plates and the inner chamber box to attain maximum thermal insulation while maintaining the desired space and structural integrity. Furthermore, in order to improve chamber settling time when the doors are opened, a two-door design with a center latch was implemented. The doors were constructed to have the same double-wall insulation design as the rest of the chamber. An optimal thickness of 4.7 mm was selected for aluminum plates meant for making the structure both rigid and lightweight.

As shown in Fig. 4, an empty space between the inner box and the bottom aluminum plate was constructed. This empty region below the inner box, called the “mixing chamber,” functions as an area where the cold air from the thermoelectric cooler is passed through the proportionally controlled heating elements. The air mixed inside this chamber is then recirculated back to the inner chamber using fan channels. Adequate air recirculation was a critical design parameter during the design phase. Based on the fluid-flow simulations, the ideal fan locations were determined and utilized. However, it proved challenging to include a DC brushless fan within a 5 cm gap between the inner chamber and the outer aluminum plates while maintaining the insulation of the chamber and the circulating air. To overcome this obstacle, 10 mm thick, space-efficient fans were mounted on 2.5 cm thick rectangular metal channels using plastic manifolds. These manifolds enabled the fans to circulate air out of the mixing chamber and into the inner chamber, allowing for adequate air flow while maintaining adequate insulation. This overall design preserved the rectangular shape of the inner chamber, thus validating the shape’s use for the models in Fig. 2 and Fig. 3.

**Fig. 5:**
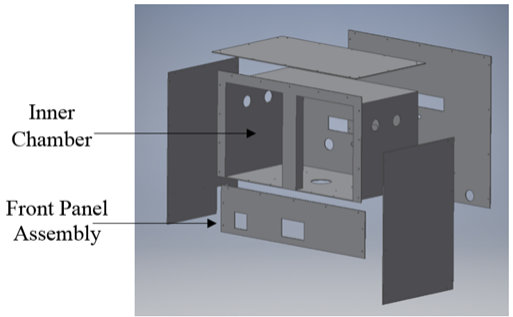
Exploded view of the temperature chamber assembly. To maintain the inner chamber’s rectangular shape, customized inserts were placed in the two slots for the doors such that, when closed, the inner wall would be a flat surface rather than contain the central protrusion. Such a protrusion, though structurally necessary, would have warranted more complicated simulations.

To provide electrical connection to resistors inside the chamber, a coaxial connector mount was designed for the back of the chamber to permit outside connections. Future plans are being formulated to automate the resistance connection process using the coaxial connection on the back of the chamber [16]. Most of the components that require regular maintenance, including control systems and electronics, were mounted on the front panel as illustrated in Fig. 5. The front panel can be easily removed without the need to disassemble the entire chamber. In the unlikely event of a system failure, system maintenance can be performed without disturbing the standard resistors inside the chamber.

## 3 Air Bath Operation

### 3.1 Control System

**Fig. 6:**
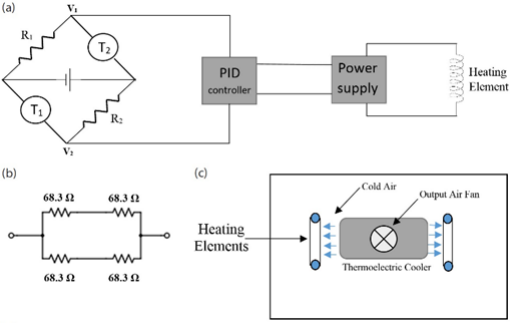
(a) The heating control system for the chamber, including a power supply coupled to a set of heating elements, a PID controller, and a set of resistors and thermistors arranged in a classical Wheatstone configuration. (b) Arrangement of the heating elements. (c) Top view of the mixing chamber.

The control system of the temperature chamber is based on the principles of the Wheatstone bridge and is shown in Fig. 6(a). Two ultrastable, low-drift thermistors (T_1_ and T_2_) with an interchangeability of ± 0.05 °C are used. These thermistors are nominally 5 kΩ at 25 °C. Using two thermistors rather than one improves the response time of the Wheatstone bridge circuit. Initially, the two thermistors were placed on the left and right sides of the mixing chamber, but during initial testing, it became evident that this thermistor configuration created a double setpoint error, causing the temperature in the chamber to periodically oscillate and to ultimately reduce the fine control of the chamber. This error was eliminated by moving the two thermistors closer together and placing them near a fan channel, with the improved configuration leading to better fine chamber control [17].

Matching wire-wound resistors are used as the R_1_ and R_2_ components of the Wheatstone bridge, so that the bridge achieves a null balance at approximately 23 °C. The system also includes a proportional-integral-derivative (PID) controller and a linear power supply that can be controlled by a voltage input of 0 V to 4 V DC [18]. Because the resistance of a thermistor is inherently a function of temperature, when the temperature deviates from 23 °C (the approximate bridge balance point), nodes V_1_ and V_2_ produce a voltage output (see Fig. 6[a]. This voltage output is used as the input for the PID controller. Based on the stored parameters in the PID controller, a voltage output between 0 V and 4 V is generated to control the linear power supply connected to the heating coils.

A low-noise heater design adopted from the previous generation of air chambers designed at NIST includes bifilar windings [17] and a linear power supply. Proportional heat is supplied to the system by using four nickel-chromium alloy heating elements that are powered by a voltage-controlled linear power supply. Four elements are carefully chosen so that the total heat power is more than the cooling power output of the thermoelectric cooler while operating within the safe margin of the linear power supply. The four 68.3 Ω heating elements are shown in Fig. 6[b] and, in our case, were wired in a series-parallel configuration such that net resistance of the entire heating element was 68.3 Ω. This configuration allowed the input current to be split between two elements and the heating elements to be mounted on both sides of the thermoelectric cooler. Each heating element was wrapped around two polytetrafluoroethylene (PTFE) posts in bifilar form on each side of the thermoelectric cooler air output as shown in Fig. 6(c).

Cold air into the chamber is provided by a 150 W air-to-air thermoelectric cooler that makes use of forced air convection provided by fans. The cooler is mounted on the bottom surface of the inner box as illustrated in Fig. 4(b) and was chosen for its compact design, DC low-noise operation, and reliable solid-state construction. Required cooling power was calculated based on the amount of heat generated by the heating system and the volume of the inner box of the air chamber. The fan mounted on the top of the thermoelectric cooler acts as an air outlet from the inner chamber. This air is then pushed through the heat sink of the thermoelectric device, allowing the air to be cooled.

The cold air passes through the heat coils inside the mixing chamber as shown in Fig. 6[c]. Fan channels inside the mixing chamber allow the air to flow back into the main chamber. The cooler in the system runs constantly at full power, and the heater element is controlled by the control system as discussed above. The thermoelectric cooler power is managed by a solid-state relay connected to the main microprocessor, allowing the automatic shutdown functionality of the cooler while the fans are kept on. The automatic shutdown function is critical in case of a system failure.

### 3.2 Microprocessor-Based System Controller with Graphical User Interface

The temperature chamber’s components and systems are monitored and controlled by a microprocessor-based controller. A Raspberry PI 3 Model B microprocessor [19] periodically measures and logs the temperature inside the chamber and provides control based on the user-selected PID parameters.

**Fig. 7:**
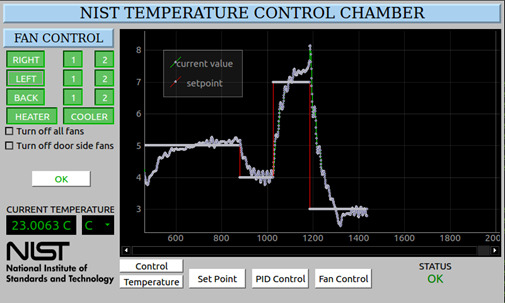
A screenshot of the system GUI. Users can interact with the fan control interface via touchscreen. The fan control buttons are shown in the upper-left corner, and parameter controls are shown on the bottom.

System control was implemented using a multichannel relay module controlled by the microprocessor. The six fans, the heater, and the cooler power are connected to a corresponding relay channel to enable separate on-off control of each device. Each relay on its corresponding module has a default closed contact. This default allows each device to be wired so that they are normally powered on to eliminate the risk of total system shutdown in the event of a microprocessor-based system controller failure. A GUI was developed to allow user-friendly interaction and complete system control using a touchscreen. Figure 7 illustrates the system controller’s main window during operation where the current value and the setpoint value are periodically measured.

### 3.3 Air-Flow Control and Safety Features

By including the fan controller feature using the relay module, users can control fans automatically using the system controller. To reduce chamber settling time, a fan control algorithm was implemented, with the fans on the corresponding door side shutting off when the doors are opened. The user has the flexibility to automatically turn off all channel fans or the door side fans when a door is opened, triggered by door sensors directly connected to the microprocessor-based system controller. The door sensors act as a switch that generates voltage signals when the door opens, prompting the microprocessor to turn off the corresponding fans based on the user configuration. See Fig. 7 for an example of the fan control menu of the GUI, where the user can control fans, heater power, and cooler power.

Safety features to protect the components and standard resistors inside the chamber were implemented in the newly designed temperature-control chamber. Due to continuous long-term use, components are expected to eventually fail, and in such cases, the chamber temperature could compromise the integrity of the standard resistors if the chamber’s internal temperature elevates to levels beyond the normal operating range. As a preventive measure, a safety mechanism was installed using the system controller and the relay board. The system controller constantly monitors the chamber temperature and overall system health. If the chamber temperature is beyond the predefined, safe operating range of the chamber, the system controller will shut off either the cooler, the heater, or both depending on the circumstances. This automated process thus protects resistance standards from temperature deviations outside the normal range of 20 °C to 25 °C.

## 4 Evaluation Methods and Data

After the final assembly of the chamber, several tests were conducted to evaluate the performance. The temperature was periodically measured and recorded using a calibrated thermometer readout with an expanded uncertainty (*k* = 2) of 0.004 °C. Subsequently, gradients and stability inside the chamber were evaluated using a 20 probe temperature scanner. Thermistor probes were placed inside the chamber in two levels, and the coordinates of each thermistor were recorded. Data gathered from these thermistors determined the temperature stability at each location inside the chamber to be approximately 0.02 °C. Furthermore, chamber settle times were evaluated when the doors were opened to determine the efficiency of the fan control mechanism described earlier.

An initial test was done to optimize sensor and component placement prior to final assembly. The obtained measurements indicated a temperature stability of approximately 0.02 °C. The limits of stability are possibly due to parameters on the PID controller that have not yet been fully optimized. Further tests were conducted to extensively evaluate the long-term stability, temperature gradients, and settle time with opened doors.

**Fig. 8:**
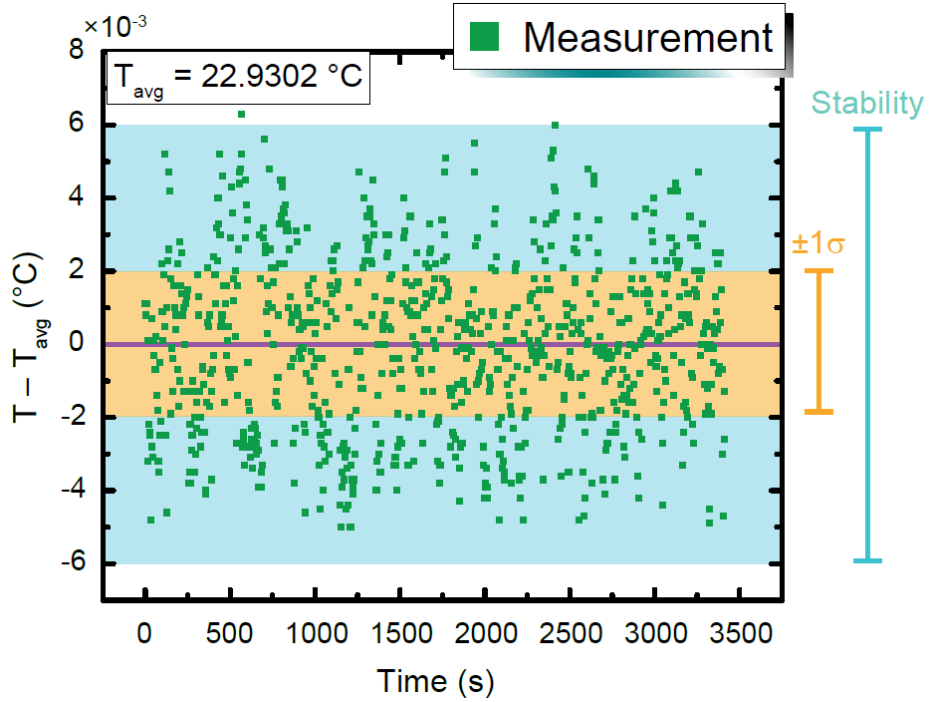
Time-dependent temperature data acquired with a thermistor readout for a period of 1 h (out of an 84 h period). The average temperature has been subtracted to more clearly gauge the level of stability observed, and the vertical axis is marked by a scaling factor of 10^−3^ at the top-left corner. The standard deviation (1σ) is 0.002 °C, and data within this range are highlighted in an orange region. The stability has been defined as approximately half of the entire observed range, which in this case yields about ± 0.006 °C.

The stability of many spatial points within the chamber was measured with the updated PID parameters, and the temperature was measured using a thermistor readout over an 84 h period. The temperature stability is illustrated in Fig. 8 with a nominal setpoint of 23 °C. The average of the temperature measurement data was 22.9203 °C, with a standard deviation of 0.002 °C. The setpoint offset was 0.08 °C, which can be easily adjusted using the GUI. These improved parameters thus resulted in a more optimized temperature environment.

The combination of simulations shown before and after the final construction both warrant additional future modifications to the mixing chamber to reduce any unused volume. Reducing unused mixing chamber volume will improve the uniformity of the air pushed into the chamber and the overall insulation of the chamber. Such improvements will be made in the near future.

## 5 Conclusions

We have reported progress on the design, development, and testing of a precise temperature-control chamber for standard resistors. With an ability to provide a constant temperature, stable to within 0.006 °C at a given point, we can exceed current-day dimensional constraints on commercially available chambers. Balancing both a large physical space with achieving temperature stability proved to be challenging, but this challenge was alleviated with the assistance of numerical air-flow simulations. Additionally, microprocessor programming allowed the air flow to be optimized within an unsealed chamber configuration to reduce chamber temperature recovery times. Tests were also carried out to verify the stability of the control system and the efficiency of the chamber.

Future plans include several modifications to accommodate pressure control and variable setpoint control for operating the chamber at different temperatures, as well as improvements on the temperature gradient bounds within the chamber as a whole. Automatic setpoint controls will also be developed and implemented. With these additional modifications, the temperature chamber can eventually be used in several other areas of calibration where precise temperature control is a requirement.
